# Geophysical upheavals and evolutionary diversification of plant species in the Himalaya

**DOI:** 10.7717/peerj.5919

**Published:** 2018-11-07

**Authors:** Kumar Manish, Maharaj K. Pandit

**Affiliations:** 1 Department of Environmental Studies, University of Delhi, Delhi, India; 2 Department of Environmental Studies, Dr. Bhim Rao Ambedkar College, University of Delhi, Delhi, India; 3 Centre for Interdisciplinary Studies of Mountain and Hill Environment, University of Delhi, Delhi, India

**Keywords:** Biodiversity, Environmental upheavals, Endemics, Himalaya, Species diversification

## Abstract

The Himalaya is one of the youngest and the loftiest mountain chains of the world; it is also referred to as the water tower of Asia. The Himalayan region harbors nearly 10,000 plant species constituting approximately 2.5% of the global angiosperm diversity of which over 4,000 are endemics. The present-day Himalayan flora consists of an admixture of immigrant taxa and diversified species over the last 40 million years. The interesting questions about the Himalayan flora discussed here are: how did the Himalaya achieve high endemic plant diversity starting with immigrant taxa and what were the main drivers of this diversity? This contribution aims to answer these questions and raise some more. We review and analyze existing information from diverse areas of earth and climate sciences, palaeobiology and phytogeography to evolve a bio-chronological record of plant species divergence and evolution in the Himalaya. From the analysis we infer the effects of major environmental upheavals on plant diversity in the region. The understanding developed in the following discussion is based on the idea that Himalaya experienced at least five phases of major geophysical upheavals, namely: (i) mega-collision between India and Eurasian plates, (ii) tectonic uplift in phases and progressive landform elevation, (iii) onset of southwest (SW) Indian monsoon, (iv) spurring of arid conditions in Central Asia, and (v) cyclic phases of cooling and warming in the Quaternary. The geophysical upheavals that were potentially disrupting for the ecosystem stability had a key role in providing impetus for biological diversification. The upheavals produced new geophysical environments, new ecological niches, imposed physical and physiological isolation barriers, acted as natural selection sieves and led to the formation of new species. This contribution aims to develop a comprehensive understanding of the plant biodiversity profile of the Himalaya in the context of complex, interconnected and dynamic relationship between earth system processes, climate and plant diversity.

## Introduction

Mountain regions comprise the large majority of the global biodiversity hotspots and it is argued that species diversification is associated with mountain building through changes in landscape and climate followed by formation of varied and heterogeneous habitats along the elevational gradients (Hoorn et al., 2013). It is equally well established that nearly all the mountains on the Earth have experienced a variety of geophysical upheavals in the geological past (Owen, 2004). For relatively younger mountain systems such as the Himalaya and Mount Kinabalu, their progress as biodiverse landscapes has also been shown to be the result of various geophysical upheavals (Pandit, Manish & Koh, 2014; Merckx et al., 2015). Formation of the Himalaya started in early Cenozoic Era around 55–50 million years ago (Mya) with the collision of the Indian and the Eurasian plates (Van Hinsbergen et al., 2012; Favre et al., 2015), an event considered as one of the greatest geophysical episodes in the Earth’s history (Harrison et al., 1992; Che et al., 2010; Wang et al., 2012). The periodic orogenic events led to physiographic and environmental changes (e.g., formation of land bridges, development of monsoon, formation of glaciers and establishment of an elaborate perennial river drainage system) and served as key drivers of the newly evolving ecosystems resulting in geographical isolation of taxa, vicariance, and evolutionary divergence of life forms (Pandit, 2017). Thus, the interconnectedness between geophysical and biological components of the Himalayan ecosystems needs to be unraveled to develop insights into understanding of the build-up of its biodiversity.

**Figure 1:**
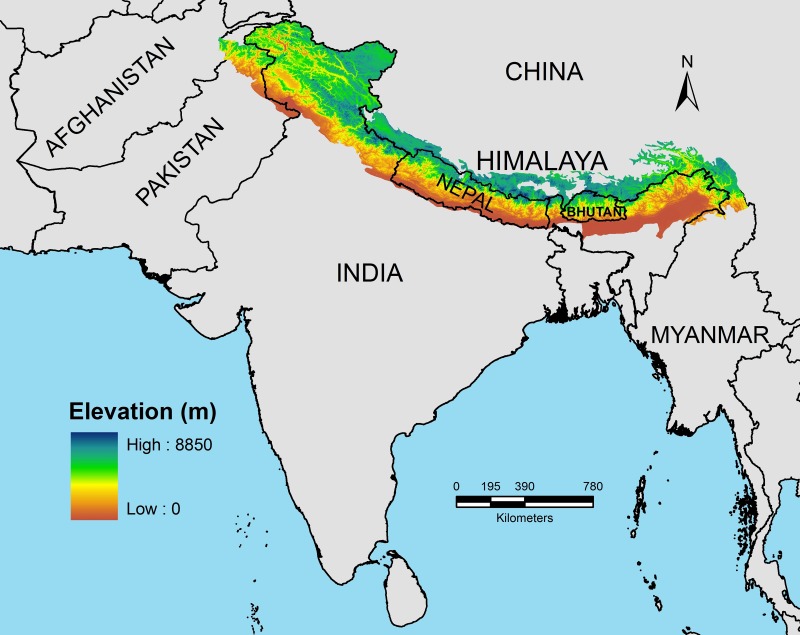
Spatial spread of the Himalayan mountain system across seven nations. The elevational gradient of the Himalaya represents the longest bioclimatic gradient of the Earth (0–8,500 m) and encompasses a myriad of ecosystems ranging from tropical, temperate and alpine. The base map was prepared using Digital Elevation Models (DEM) in Arc GIS 9.3 sofware (Environmental Systems Research Institute (ESRI), Redlands, CA, USA).

The Himalaya encompasses a geographical area of nearly 3.4 million km^2^ and is spread across nations of Afghanistan, Pakistan, India, Nepal, China (Tibetan Autonomous Region), Bhutan, and Myanmar (Pandit, Manish & Koh, 2014; Fig. 1). Geographically, the Himalaya extends from Namcha Barwa mountain range in India’s east to Nanga Parbat massif in the west forming an arc of about 2,400 km (Fig. 1). Geologically, Himalaya is divided into four distinct litho-tectonic and physiographic units from north to south, namely Outer Himalaya or Siwaliks, Lesser Himalaya, Greater Himalaya, and Trans-Himalaya (Valdiya, 2002). The average elevational range of Siwaliks is 900–1,500 m, followed by the Lesser Himalaya with an average elevational range of 500–2,500 m. The Greater Himalayan elevations range from 6,000–7,000 m and the northernmost Trans-Himalaya (Hedin, 1909) mostly comprises plateau areas to the north of the Indus and Brahmaputra rivers with average elevation of 5,000–6,000 m (Valdiya, 2002; Pandit, 2017). Eco-climatically, the Himalaya is broadly classified into Eastern and Western Himalaya. The Eastern Himalaya (EH) stretches from 21°–25°N latitudes across the east of Kali Gandaki valley encompassing eastern Nepal, north-eastern Indian states of Sikkim, Arunachal Pradesh and the hill areas of North Bengal, Bhutan, and northern Myanmar. The Western Himalaya (WH) extends from 30°–40°N latitudes across the west of Kali Gandaki valley encompassing western Nepal, Indian states of Uttarakhand, Himachal Pradesh, Jammu and Kashmir and areas of northern Pakistan and Afghanistan (Fig. 1). The EH region experiences heavy annual average rainfall of 3,800–4,000 mm while the WH is comparatively drier with an annual average rainfall of 75–150 mm (see Pandit, 2017). The rainfall plays a major role in determining the east–west bioclimatic gradient of the Himalaya. Some authors have designated a part of the mountain range as the Central Himalaya (Singh & Singh, 1987; Singh, Adhikari & Zobel, 1994; Vetaas, 2000). CH extends from river Kali in the east to river Tons (largest tributary of Yamuna river) in the west encompassing central Nepal and central Uttarakhand (India).

The total number of higher plant species in the Himalaya varies from 8,000–10,000 with about 40% of these taxa as endemics (Pandit, Manish & Koh, 2014). It is well known that majority of the Himalayan flora consists of immigrated plant taxa that have evolved and diversified over millions of years following the Himalayan formation (Singh & Singh, 1987; Pandit & Kumar, 2013; Pandit, Manish & Koh, 2014; Manish, 2017). It is, therefore, of significant interest to evolutionary biologists as to how starting with an immigrant flora, the Himalaya now harbors such a high number of plant endemics. This question, though fascinating, has not been much investigated or discussed in ecological literature. To address this knowledge gap, we need to develop an understanding of the intricate relationship between geodynamic processes of the Himalayan mountain building and its varied biodiversity gradients. To the best of our knowledge, there are only limited studies that have attempted to understand this relationship in an integrated manner (Pandit & Kumar, 2013; Pandit, Manish & Koh, 2014; Favre et al., 2015; Pandit, 2017). The large majority of studies on the Himalaya have mostly focused on the evolutionary consequences of a specific geological period (Miao et al., 2012) or concentrated on a specific geographic region (Wen et al., 2014; Favre et al., 2015). Hence, a broader and a more comprehensive understanding of the evolutionary diversification of Himalayan flora is warranted. In this contribution, we seek to address this knowledge gap by evolving a sequence of plant species divergence episodes during major geological periods in the Himalaya and identifying their relationship with the environmental changes in the region. An overarching goal of this study was to analyze the existing published information on Himalayan plant diversity and understand the intricate relationship between the build-up of plant diversity and physical-climatological variations produced by the geophysical changes during various phases of the Himalaya’s formation.

## Survey Methodology

We used four standard databases, namely Web of Science (http://www.webofknowledge.com), Google Scholar (https://scholar.google.co.in/schhp?hl=en), Science Direct (http://www.sciencedirect.com), and PubMed (http://www.ncbi.nlm.nih.gov/pubmed) to systematically identify peer-reviewed journal and book articles using a combination of controlled vocabulary and free text terms based on the following keywords and terms: “Himalaya” AND “Arid”, “Himalaya” AND “Biogeography”, “Himalaya” AND “Ecology”, “Himalaya” AND “Evolution”, “Himalaya” AND “Formation”, “Himalaya” AND “Fossil”, “Himalaya” AND “Glacier”, “Himalaya” AND “Gondwana”, “Himalaya” AND “Ice Age”, “Himalaya” AND “Monsoon”, “Himalaya” AND “Paleo”, “Himalaya” AND “Plants”, “Himalaya” AND “Refugia”, “Himalaya” AND “Tectonic” and “Himalaya” AND “Uplift”. All search fields were considered in the database while searching. Articles were searched for all periods up to, and including, December 2017 in English language irrespective of the number of citations. We ensured that we covered all the peer-reviewed articles that included the term “Himalaya” anywhere in the text, instead of just in the title, abstract or keywords. The resulting list of articles was then screened for whether the study included plant or animal species and the studies dealing with the latter (animal species) were largely excluded from further consideration (unless critical to the discussion). Additionally, we also excluded studies that were not published in peer-reviewed scientific conferences and conference proceedings. To increase the scope and coverage of the present review, we also applied a snowball search technique (Greenhalgh & Peacock, 2005) where we made a manual search for published peer-reviewed studies in the respective references of the selected publications and then included all studies in the present review that matched the above keywords and terms. For each selected publication, we retrieved the following information: author name (s), title, year of publication, journal title, sampling area and studied species.

## Geological Backdrop of the Himalaya

Indian continent was once a part of Gondwanaland—a supercontinent formed nearly 600 Mya (Murphy et al., 2008). Gondwanaland covered much of the Southern Hemisphere comprising the present day South America, Africa, Madagascar, Seychelles, India, Australia, and Antarctica. Gondwanaland split around 180 Mya as a result of sea-floor spreading and development of a series of oceanic deep-seated mantle plumes resulting in Western Gondwana (comprising Africa and South America) and Eastern Gondwana (comprising Madagascar, India, Seychelles, Australia and Antarctica). Around 120 Mya, the split of Western Gondwana led to separation of South America from Africa, and another fragment containing India-Madagascar-Seychelles (IMS) separated from Antarctica and Australia (Chatterjee & Scotese, 1999). The newly separated IMS fragment migrated northward across the Tethys ocean towards the Eurasian continent at varying speeds ranging between 5–40 cm/year (Jagoutz et al., 2015). The drifting IMS fragment carried along a host of primitive flora of Gondwanan origin such as seed ferns (*Glossopteris*, *Dicroidium*, *Sphenobaiera*, *Linguifolium*), conifers (*Heidiphyllum*, *Voltziopsis*, araucarians and podocarps) and lycopods (*Cyclomeia*) (McLoughlin, 2001). Widespread seafloor spreading around 80–90 Mya further widened the central Indian Ocean and resulted in detachment of the Madagascar block from the IMS fragment (Plummer & Belle, 1995). The Indian-Seychelles plate that drifted at ∼5 cm/year suddenly tripled its speed to ∼15 cm/year, the fastest recorded migration speed for any tectonic drift in the geological history (Jagoutz et al., 2015). During its northward traverse around 65 Mya, the Deccan flood basalts erupted and repositioning of the western Indian Ocean spreading ridge occurred that led to the separation of Seychelles from the Indian plate (Duncan & Pyle, 1988; McLoughlin, 2001). Subsequently, the Seychelles block stationed close to Africa while India continued migrating northward. Around 55–50 Mya (Early Eocene), the drifting Indian plate collided with Eurasia along the northeastern corner of Greater India with the collision progressing westwards until 40 Mya (Van Hinsbergen et al., 2012; Bouilhol et al., 2013; Favre et al., 2015). The India-Eurasia collision led to extensive deformation of the northern margin of Indian plate and a major portion of the Indian plate subducted underneath the Asian plate. The collision also led to the draining of the Tethys Sea and upliftment of the long settled Tethyan geosyncline coastal sediments as meta-sedimentary formations. The continental collision also laid the foundation of the youngest and loftiest mountain system of the world—the Himalaya.

## The Drifting Indian Plate: Raft or an Island?

From the above account, it is reasonable to guess that post separation from Gondwanaland, the Indian plate may have been an isolated island continent for nearly 45 million years, which could have created conditions for the evolution of a high endemic biodiversity. The fossil record of India, however, provides equivocal evidence on the extent of pre-Himalayan biotic endemism (see Pandit, 2017). Fossil records belonging to the Upper Cretaceous to Lower Tertiary in the Deccan Intertrappean beds of Southern India reveal a mixed flora with wide geographical affinities ranging from disparate regions, namely Africa (*Palmocaulon hyphaeneoides*, *Palmoxylon hyphaenoides*), Australia (*Eucalyptus dharmendrae*, *Tristania confertoides*), Madagascar (*Palmoxylon ghughuense*), and South America (*Rodietes*, *Cyclanthodendron*) (see Srivastava, 2011; Pandit, 2017). Presence of fossil taxa with such varied geographical affinities indicates the likelihood of biotic exchanges between these disjunct landmasses and also that India may not have been isolated in strict sense to induce high endemism (Pandit, 2017). Isolated or connected, some researchers have reported presence of high endemic biodiversity on the Indian plate that likely developed during 45 million years of its isolation (see Srivastava, 2011). As such, much of the diversity of Indian plate was decimated due to eruption of Deccan volcanoes around the Cretaceous-Tertiary boundary with bulk of these eruptions occurring in the early Paleocene between 67–65 Mya (Officer et al., 1987; Khosla & Sahni, 2003). Notwithstanding these catastrophic events, many ancient Gondwanan lineages did manage to survive and disperse into Asia when India collided with Eurasia (Bossuyt & Milinkovitch, 2001). The proponents of “out-of-India” hypothesis have referred to the Indian plate as a ‘raft’ for ferrying a number of taxa from Gondwanaland to mainland Asia (Bossuyt & Milinkovitch, 2001; Karanth, 2006). The “out-of-India” hypothesis has received support from investigations of the plant family Crypteroniaceae suggesting that the family originated in west Gondwanaland and subsequently reached Asia by rafting on the Indian plate (Conti et al., 2002). Recent fossil leaf impression data from genus *Alphonsea* (Annonaceae) from the Tertiary sediment deposits of Assam suggests that the genus originated in India during Late Oligocene and migrated to South East Asia via Myanmar during Early Miocene (Srivastava & Mehrotra, 2013).

## The First Migration Wave

An immediate consequence of the collision of the Indian and Eurasian plates was the establishment of a contiguous landmass connecting Indian Peninsula with the Sino-Japanese regions in the north and the Malayan Archipelago in the southeast (Pandit, Manish & Koh, 2014). This landmass connectivity was the result of cessation of marine deposition and the beginning of terrestrial sedimentation in the suture zone of India-Eurasia collision (Mehrotra et al., 2005). A biological vacuum was created in the erstwhile nascent Himalayan ecosystems as a result of extinctions caused by Cretaceous-Tertiary volcanism event. This vacuum was gradually filled by large-scale floral migrations from the adjacent connected regions in the east, north and south (Singh & Singh, 1987). Thus, the newly evolved Himalayan landscape started to serve as a ‘intercontinental biological highway’ for migrating flora from all directions (Pandit & Kumar, 2013; Pandit, Manish & Koh, 2014).

The first taxa to colonize the Himalayan landforms were the ones with tropical affinities such as Alangiaceae, Dipterocarpaceae, Ebenaceae, Ericaceae, Gleichneaceae, Rhamnaceae, Malvaceae and Sapotaceae since climatic conditions in the region were essentially tropical in nature at the time of collision (Mehrotra et al., 2005). Faced with no dispersal barriers, either oceanic or climatic, these taxa found novel opportunities to colonize and intermingle in the newly formed Himalayan landmasses with numerous unoccupied niches. Majority of the early migrants which first crossed through the northeastern route (via present day Arunachal Pradesh) were the ones with largely Sino-Japanese and Malayan affinities such as *Dalbergia*, *Dipterocarpus*, *Lagerstroemia*, *Myristica*, *Pittosporum*, *Shorea*, and *Terminalia* (Singh & Singh, 1987). Fossil records of genera such as *Anisoptera*, *Dipterocarpus*, *Hopea* and *Shorea* (Dipterocarpaceae), *Zizyphus* (Rhamnaceae), and *Diospyros* (Ebenaceae) are abundant in the deposits of northeast India belonging to the Middle Miocene epoch (see Srivastava & Mehrotra, 2010 and references therein). More importantly, no fossil records of these taxa appear anywhere in India during the entire Paleogene Period, but are reported to dominate the fossil deposits found from Middle Miocene onwards. This first phase of plant migration lasted for almost 30 million years and it gradually stopped when connections were lost due to the uplift of Himalaya with a dissected topography as a result of climatic and morpho-tectonic changes in the subsequent epochs (Pandit, 2017).

## Uplift of the Himalaya

Formation of the Himalaya and other mountain ranges in Tibet and farther north, such as Qinling, Taihang, Hengduan, and Tianshan started around 45 Mya and continues till now (see Wadia, 1957; Pandit, 2017). There are two contrasting views in literature regarding the timing and sequence of the uplift of the Himalaya. The first one holds that the Himalaya started to rise against a pre-existing proto-Tibetan highland that was already as high as 4,500 m since at least 45 Mya (Ding et al., 2017; Spicer, 2017). The Himalaya have continued to rise in a phased manner against this proto-Tibetan highland, attaining elevations of 1,000 m around 56 Mya, 2,500 m around 23 Mya, 4,000 m around 19 Mya and 5,000 m around 15 Mya (Ding et al., 2017; Spicer, 2017). It was around 15 Mya that Mount Everest came into existence and the average height of the Himalaya became greater than the height of the Tibetan Plateau (Spicer, 2017). Subsequently, the Himalaya continued to rise another 3,000 m due to renewed and considerable tectonic activity in Pleistocene around 3–2.5 and 0.98 Mya (Spicer et al., 2003; Spicer, 2017). The second view of the Himalayan mountain building proposes that the Himalaya along with Tibet rose as one block in a phased manner (Harrison et al., 1992; Molnar, England & Martinod, 1993; Favre et al., 2015). According to this view, four major episodes or windows of uplift have been reported for the Himalaya, namely 45–35 Mya, 35–20 Mya, 20–10 Mya, and 8–6 Mya (Wen et al., 2014; Favre et al., 2015). Notably, the phased elevation episodes were characterized by distinct sets of geophysical developments: 45–35 Mya was characterized by the subduction of Indian plate under the Eurasian plate; 35–20 Mya represented the period during which the Himalaya attained average elevation of 4,000 m and the modern-day southwest (SW) Indian monsoon began to take shape; 20–10 Mya period was accompanied by arrival of wet summer period south of the Himalaya and gradual aridification of the central Asian region to the north; 8–6 Mya represented the period of the major Himalayan uplift at its eastern edge (mostly Tibetan plateau) followed by intensification of monsoon (Zhisheng et al., 2001; Spicer et al., 2003; Wen et al., 2014; Favre et al., 2015).

Despite the equivocal evidence on the phased mode of the Himalaya’s elevation, it is certain that the Himalayan uplift caused three major environmental changes in the region: (i) land connections formed in the preceding epochs between the adjacent landmasses of India, Sino-Japanese and Southeast Asia were lost, (ii) the range of elevational climatic gradient in the Himalaya extended from tropical to temperate and alpine, and (iii) an orographic barrier was formed resulting in the formation of the SW monsoon system (Pandit, 2017). With the development of a cooler temperate environment towards higher elevations, new opportunities arose for the immigration of a number of temperate elements from the Sino-Japanese, European, and Mediterranean regions. Thus, started a second wave of immigration into the Himalaya in which many temperate taxa such as *Acer*, *Alnus*, *Betula*, *Desmodium*, *Meliosma*, and *Quercus* (Sino-Japanese affinities) found their way into the Himalaya from the northwestern end (via present day Jammu & Kashmir) (Mehrotra et al., 2005). Other examples of the immigrant taxa into the Himalaya from different regions include: *Anemone*, *Caltha*, *Clematis*, *Ranunculus* and *Viola* (European, Russian, and north Asian), *Fagonia* (Egyptian), *Melilotus* (European and Siberian), *Seseli* (Russian and Siberian), and *Trifolium* (European, Siberian, and North African) (Mani, 1974). Some researchers have suggested the formation of a “Himalayan corridor” to the south of Great Himalaya and its essentially temperate nature (Kitamura, 1955). It was through this corridor that numerous plant taxa of Sino-Japanese origin migrated westwards and southwards into the Himalaya (see Pandit, 2017). The formation of the “Himalayan corridor” has been confirmed in a number of later studies (Wang, 1992; Sun, 2002; Tabata, 2004).

## Onset of Monsoon and Miocene Biodiversity

The onset of SW monsoon system triggered when the average elevation of the Himalaya reached about 4,000 m by the end of Oligocene (28–23 Mya) (Rowley, Pierrehumbert & Currie, 2001; Favre et al., 2015). However, the exact timing of the initiation of monsoon is a matter of much debate. Some researchers argue that the monsoon system originated as early as in Eocene (Srivastava et al., 2012; Shukla et al., 2014; Renner, 2016) or Paleocene epoch when Indian plate reached the Tropic of Capricorn (Patnaik et al., 2012). Irrespective of the timing of its initiation, it is certain that the monsoon system assumed its present-day form (with respect to seasonality and intensity) only by the end of Oligocene and beginning of Miocene when the elevated Himalaya began to act as an orographic barrier to the flow of regional winds in the west-east direction (Favre et al., 2015; Pandit, 2017). Around 8–6 Mya, an extensive uplift of the Tibetan plateau occurred which apparently intensified the monsoonal system (Burbank, Derry & France-Lanord, 1993). Sedimentological records available from the Siwalik foredeep corroborate the intensification of Asian monsoon at 6 Mya, with a prominent peak at 5.4 Mya (Sanyal et al., 2004).

The monsoon played a decisive role in the landscape evolution of the Himalaya. The ensuing heavy rainfall along the frontal ranges of the Himalaya gave rise to numerous streams and rivers which transported vast quantities of sediments and regularly denuded the exposed rock surfaces leading to the formation of deeply incised valleys (Pandit, 2017). The landscape incision was more prominent in the EH than the WH because of its tropical latitudes ensuring prolonged and more intense period of rainfall (Bookhagen, Thiede & Strecker, 2005). As a result, the EH region was transformed into a “region of extreme relief” due to closely clustered high, steep mountains that were dissected by numerous river divides and deep valleys (Irving & Hebda, 1993). The monsoon, therefore, was key to transforming the Himalaya into a highly dissected landscape aided by the tectonic processes that accelerated weathering, denudation and sediment transport driven mainly by precipitation (Pandit, 2017). Differentiated into numerous valleys dissected by rivers, the fragmented Himalayan landscape after the uplift provided novel physical and physiological barriers for gene flow between once continuous plant populations (Favre et al., 2015; Zhao et al., 2016). The combined result of these geographic events and formation of geographic barriers to gene flow culminated in large-scale allopatric speciation and evolutionary diversifications of the Himalayan flora (Pandit, Manish & Koh, 2014; Favre et al., 2015). Various molecular phylogenetic studies provide evidence that many plant species complexes (*Caragana*, *Cyananthus, Koenigia, Meconopsis, Rheum* and *Rhodiola*) originated and diversified in the period after the onset and intensification of modern-day monsoon system in the Himalaya (see Table 1). However, Renner (2016) argued that most of the time-calibrated molecular phylogenetic studies that link clade age of species between 15–0.5 Mya with specific uplift phases of the Tibetan plateau and its associated environmental effects assume that the Tibetan plateau underwent most major uplift during the Miocene, whereas the Tibetan Plateau had already reached the height of 4,000–5,000 m in Paleogene (∼40 Mya). Extensive weathering, erosion and detritus transport along the Himalayan slopes due to monsoon also aided in the formation of a foreland basin and transformation of the vegetation profile of the Himalayan from tropical wet evergreen forests of C3 plants to tall grassland ecosystems comprising predominantly of C4 plants around 8–6 Mya (Hoorn, Ohja & Quade, 2000). This shift from C3 to C4 dominated ecosystem was due to widespread global environmental changes in the Late Cenozoic Era such as global cooling and significant drop in global carbon dioxide (CO_2_) levels due to increased rates of chemical weathering and trapping of CO_2_ in the ocean sediments (Raymo & Ruddiman, 1992; Quade & Cerling, 1995). It has, however, been reported that the predominance of C4 plants in the Himalayan ecosystems has declined since the Last Glacial Maximum due to increased CO_2_ and humidity levels in the atmosphere (Galy et al., 2008).

**Table 1:** Molecular phylogenetic studies that have related plant species diversifications and evolution to specific phases of the Himalayan mountain formation and its uplift. The studies have been listed in an alphabetical order according to the author names and year of publication.

**Clade(Family)**	**Crown age of clade (Mya)**	**Methodology**	**Principal Findings**	**Source**
*Koenigia* (Polygonaceae)	13.72–4.91	MD and DT	Uplift of the Himalaya promoted species diversification in *Koenigia*; Himalaya acts as a primary evolutionary centre of *Koenigia*	Fan et al. (2013)
*Hippophae tibetana* (Elaeagnaceae)	4–1	MD and DT	Strong allopatric divergence was promoted in *Hippophae tibetana* during the Last Interglacial period (0.13–0.115 Mya) by orogenic processes and climate oscillations during the Quaternary	Jia et al. (2011)
*Spiraea alpina* (Rosaceae)	1.2–0.6	MD and DT	Uplift of the Tibetan Plateau and severe climatic oscillations during Quaternary promoted intraspecific divergence of *Spiraea alpina*	Khan et al. (2014)
*Nannoglottis* (Asteraceae)	1.94–1.02	MD and DT	Uplift of the Tibetan Plateau and severe climatic oscillations during Quaternary led to origin of several species of *Nannoglottis*	Liu et al. (2002)
*Ligularia*-*Cremanthodium*-*Parasenecio* (Asteraceae)	13–8	MD and DT	Uplift of the Tibetan Plateau between Early Miocene to Pleistocene promoted rapid and continuous allopatric speciation in the *Ligularia-Cremanthodium-Parasenecio* complex	Liu et al. (2006)
*Taxus wallichiana* (Taxaceae)	6.5–2.0	MP and SDM	Diversification and evolution of *Taxus wallichiana* in the Himalaya was promoted by Miocene/Pliocene geological and climatic events, uplift of the Tibetan Plateau and Late Quaternary climatic oscillations	Liu et al. (2013)
*Ostryopsis intermedia* (Betulaceae)	1.2–0.5	DT and ENM	Climatic oscillations during Quaternary and uplift of the Tibetan Plateau caused hybrid speciation of *Ostryopsis intermedia*	Liu et al. (2014)
*Dasiphora* (Rosaceae)	3.25–0.32	DT and DET	Uplift of the Tibetan Plateau and severe climatic oscillations during Quaternary caused deep divergences in *Dasiphora*	Ma et al. (2014)
*Rheum* (Polygonaceae)	4.2–3.6	MD and DT	Extensive uplifts of the Tibetan Plateau promoted diversification of species in *Rheum*	Sun et al. (2012)
*Fagopyrum tibeticum* (Polygonaceae)	14.8–6.4	MD and DT	Uplift of the Tibetan Plateau led to species radiation and development of woodiness in *Fagopyrum tibeticum*	Tian et al. (2011)
*Rheum* (Polygonaceae)	7	MD and DT	Uplift of the Tibetan Plateau coupled with climatic oscillations in the Quaternary led to adaptive radiation in *Rheum*	Wang, Yang & Liu (2005)
*Dolomiaea* (Asteraceae)	13.6–12.2	MD and DT	Uplift of the Tibetan Plateau since Miocene led to the evolution of endemic Himalayan flora	Wang, Liu & Miehe (2007)
*Hippophae tibetana* (Elaeagnaceae)	3.15–1.04	MD and DT	Rapid uplift of the Tibetan Plateau affected the dispersal potential and species differentiation of *Hippophae tibetana*	Wang et al. (2010)
*Pomatosace filicula* (Primulaceae)	2.66–0.73	MD and DT	Divergence in *Pomatosace filicula* overlaps with the Quaternary glaciation history in the Tibetan Plateau in the Early and Middle Pleistocene	Wang et al. (2014)
*Meconopsis* (Papaveraceae)	15–11	MD and DT	Divergence of *Meconopsis* was driven by the uplift of the Tibetan Plateau	Xie et al. (2014)
*Meconopsis integrifolia* (Papaveraceae)	7.86–3.45	MD and DT	Uplift of the Tibetan Plateau and associated climatic changes triggered the initial divergence of *Meconopsis integrifolia*	Yang et al. (2012)
*Isodon* (Lamiaceae)	26.44–14.66	MD and DT	Uplift of the Tibetan Plateau and associated climatic changes led to rapid radiation of *Isodon*	Yu et al. (2014)
*Caragana* (Fabaceae)	16–14	MD and DT	Uplift of the Tibetan Plateau and onset of the Himalayan motion led to high evolution and diversification of *Caragana*	Zhang & Fritsch (2010)
*Stellera chamaejasme* (Thymelaeaceae)	6.5892	MD and DT	Uplift of the Tibetan Plateau and associated climatic changes led to the origin of *Stellera chamaejasme*	Zhang, Volis & Sun (2010)
*Soroseris-Stebbinsia-Syncalathium* (Asteraceae)	8.44–1.56	MD and DT	Uplift of the Tibetan Plateau and associated changes in climate and habitat fragmentation led to rapid diversification and radiation of *Soroseris-Stebbinsia-Syncalathium*	Zhang et al. (2011)
*Phyllolobium* (Fabaceae)	3.96–3.48	MD and DT	Uplift of the Tibetan Plateau in the Late Pliocene and Early-to-Mid Pleistocene along with Late Pleistocene Glaciation led to rapid diversification of *Phyllolobium*	Zhang et al. (2012)
*Rhodiola* (Crassulaceae)	21	MD and DT	Uplift of the Himalaya and onset of Himalayan motion led to origin of *Rhodiola*	Zhang et al. (2014)
*Cyananthus* (Campanulaceae)	23–12	MD and DT	Onset of the Himalayan motion led to the origin of *Cyananthus*	Zhou et al. (2013)

**Notes.**

Mya million years ago MD Molecular dating DT Divergence time analysis MP Molecular phylogeography SDM Species distribution modelling ENM Ecological niche modelling DET Demographic test analysis

Megafossil evidences including leaf impressions, wood elements, flowers and fruits indicate that until the end of the Oligocene and beginning of Miocene epochs, the uplifting Himalayan landforms were dominated by tropical Indian peninsular flora such as *Ficus* sp., *Mesua ferrea*, *Mallotus philippensis*, *Kayea floribunda*, *Chukrasia tabularis*, etc. (see Srivastava et al., 2014). Presence of tropical and humid conditions during Early Miocene epoch has also been confirmed by the discovery of *Ficus palaeoracemosa* from the Kasauli geological formation (age: 23–10 Mya) in WH (Srivastava, Srivastava & Mehrotra, 2011). During Mid-Miocene when the Himalaya attained an average elevation of 2,200–2,400 m, many sub-tropical and temperate floral elements migrated into the Himalaya. This was revealed by the discovery of *Trachycarpus* and *Prunus* from the Miocene sediments of the Ladakh-Karakoram region (Guleria et al., 1983; Lakhanpal et al., 1984; Srivastava et al., 2014). Forests around this time (Mid-Miocene) at higher elevations had numerous temperate taxa such as *Alnus*, *Picea*, *Pinus* and *Betula* (Singh & Singh, 1987; Phadtare, 2000). The palynological records from the Surai Khola region of Central Nepal reveal that grasslands with predominantly C4 plants dominated the Himalayan foothills from Late Miocene to Pliocene and Early Pleistocene (Hoorn, Ohja & Quade, 2000).

Climate-driven landscape changes resulted in alterations in plant physiology, and geographic isolation and speciation through vicariance. Once these species complexes originated and diversified in the Himalaya, these subsequently dispersed into the neighboring regions of Asia Minor, Central Asia, Mongolian plateau and Europe producing derivative species therein (Jia et al., 2012; Nie et al., 2013). Ohba (1988) proposed the term “Central Asiatic highland corridor” through which the flora originating in the Himalaya migrated northward to the Central Asian highlands. Examples of such emigrant taxa from the Himalaya are: *Anaphalis* (migrated to eastern Asia, South East Asia and North America) (Nie et al., 2013), *Hippophae rhamnoides* (migrated to Central Asia and Asia Minor) (Jia et al., 2012), *Lagotis* (migrated to Central Asia and Arctic highlands) (Li et al., 2014), *Leontopodium* (migrated to European mountain ranges) (Blöch et al., 2010), *Rhodiola* (migrated to Northern Hemisphere regions such as Europe and Central Asia) (Zhang et al., 2014) and *Solms-laubachia* (migrated to Hengduan Mts) (Yue et al., 2009). The advent of the monsoon system is believed to have led to the formation of a mesic corridor in the Indian sub-continental region through which plant species such as *Begonia* migrated from Africa to Southeast Asia via the Himalaya (Rajbhandary et al., 2011).

## Aridification of Central Asia

Following the development of SW monsoon system, aridity in the Central Asian region increased due to blockage of moisture laden winds by the uplift of the Himalaya and the Tibetan Plateau (Miao et al., 2012). The aridification triggered the diversification of many plant species with xerophytic adaptation and colonization of the Tibetan Plateau. Specific examples of species divergence include split of arid palmate *Frutescentes* section from its sister clades in the *Caragana* species complex around 8–7 Mya (Zhang & Fritsch, 2010). Similarly, *Phyllolobium*, a genus diversified as a result of intense uplift, cold climate, and ensuing aridity in the Tibetan Plateau (Table 1; Zhang et al., 2012). Central Asian aridification is also reported to have triggered the divergences of three lineages of *Ephedra* (eastern Tibetan Plateau, southern Tibetan Plateau, and northern China) (Qin et al., 2013). Likewise, the dominance of *Artemisia* in the present day vegetation of southwest and southeast regions of Tibetan Plateau is the result of rapid uplift of Tibetan Plateau and consequent prevalence of dry climate during and after the Late Miocene (Yunfa et al., 2011).

## Quaternary Climate and the Himalayan Biodiversity

The Quaternary Period starting 2.6 Mya was characterized by expansive climatic fluctuations including repeated advance and retreat of glaciers with intermittent warming stages (Owen, Finkel & Caffee, 2002; Owen, 2009). Almost the entire Northern Hemisphere including Europe, North America and North-west Asia witnessed widespread glaciations with ice sheets descending as far south as to 40°N latitude (Carlson & Winsor, 2012). However, in the Himalaya, glaciations were confined to mountain peaks and high elevations (Owen, 2009). More importantly, unlike other regions of the world, there is no evidence of a uniform ice sheet covering the entire Himalayan range during the Quaternary glacial stages (Owen, 2009). In fact, most of the glaciers are reported to have advanced up to 10 km from their present-day ice margins during various stages of glaciation (Owen, Finkel & Caffee, 2002; Owen, 2009). Thus, the extent of glaciations in the Himalaya has been rather restricted as compared to the other mountain systems such as Alps and Andes, most likely due to the tropical location of the mountain range (Pandit, 2017).

Overall, 3–4 significant glacial advances and retreats have been documented in different regions of the Himalaya during the Quaternary period: Tibetan Plateau (4), Zanskar ranges (3), Swat valley (3), Kanchenjunga ranges (4) and Khumbu mountains (4) (see Owen, Finkel & Caffee, 2002 and references therein). Despite the limited extent of glaciation, the advance and retreat of glaciers had a significant effect on the evolutionary diversification of plant species in the Himalaya (Pandit, 2017). Some species got extinct, some dispersed to new warmer habitats in the south while some survived in glacial refugia in non-glaciated habitats. Habitats with patchy landscape distribution and diverse environmental conditions offered spaces as refugial habitats for numerous plant species. Examples of refugial habitats include low elevation areas of Tibetan Plateau (*Aconitum gymnandrum*, *Juniperus tibetica*, *Primula secundiflora*), south-eastern edge of Tibetan Plateau (*Potentilla fruticosa*, *Metagentiana striata*), and neighbouring Hengduan Mts (*Pedicularis longiflora*, *Tsuga dumosa*, *Juniperus przewalskii*, *Sinopodophyllum hexandrum*) (Zhang et al., 2005; Chen et al., 2008; Wang, 2008; Yang et al., 2008; Li et al., 2009; Wang et al., 2009; Cun & Wang, 2010; Opgenoorth et al., 2010; Li et al., 2011; Hoorn et al., 2013). Moreover, multiple refugia for a single plant species have also been reported such as for *Pomatosace filicula* (Wang et al., 2014), *Hippophae neurocarpa* (Kou et al., 2014), *Hippophae tibetana* (Jia et al., 2011), *Rhododendron simsii* (Li, Yan & Ge, 2012) and *Aconitum gymnandrum* (Wang et al., 2009). Development of multiple refugia was facilitated by the steep elevational gradient of the Himalaya that allowed various taxa to rapidly disperse to lower elevation habitats after traversing short geographic distances (Wen et al., 2014). Multiple recolonization events from numerous refugia have been reported after the glacial retreat (Cun & Wang, 2010; Wen et al., 2014; Meng et al., 2015). As a result of episodic glacial advances and retreats, the geographic ranges of the refugial plant taxa underwent repeated contractions and expansions. Consequently, the multiple recolonization events resulted in frequent mixing of floral taxa from different refugia followed by periodic hybridizations, adaptive radiations and speciation resulting in greater species diversity (Pandit, 2017). Hybridizations post recolonization events from refugia have been cited as an important mechanism for the colonizing success of *Rhododendron* (Zha, Milne & Sun, 2008; Milne et al., 2010), *Meconopsis* (Yang et al., 2012), and *Pinus densata* (Gao et al., 2012). Earlier studies have reported that when previously isolated populations come in contact with each other due to large scale glacial dynamics, hybridization followed by polyploidy is a common phenomenon resulting in species divergence (Stebbins, 1984). There is evidence of colonization success and dominance of polyploids in post-Pleistocene plant taxa in the Himalaya (Pandit, Manish & Koh, 2014; Pandit, 2017).

Pollen data from the Pleistocene epoch of Uttarakhand region in the WH indicates that evergreen oaks (*Quercus semecarpifolia*) and alder (*Alnus*) dominated the Himalayan landscapes around 0.0078 Mya when climate conditions were cold and wet with moderate monsoon and were subsequently replaced by conifers (*Pinus* and *Abies*) around 0.0066 Mya when climate became warmer (Phadtare, 2000; Pandit, 2017). This indicates that repeated climatic oscillations in the Quaternary influenced the vegetation composition of the Himalaya. It has also been postulated that during each glaciation phase, the temperate and alpine flora of the Himalaya moved southwards towards the lower elevations where warmer temperatures prevailed (Mani, 1974; Pandit, 2017). During the interglacial phases with the retreat of glaciers, these taxa subsequently became isolated and diversified. This is evident by the common occurrence of large number of plant species with Sino-Himalayan plants affinities on isolated hilltops of peninsular India, Western and Eastern Ghats and as far south as Sri Lanka. Some examples of such temperate taxa representing Pleistocene relicts are: *Anemone rivularis* (Himalaya, Nilgiri and Palni Hills), *Clematis wightiana* (Himalaya, Nilgiri, Shevaroy and Palni Hills), *Cnicus wallichii* (Himalaya, Nilgiri and Palni Hills), *Geranium nepalense* (Himalaya, Khasi Hills, Nilgiri and Sri Lanka), *Gymnopetalum* (Deccan, Chotta Nagpur plateau), *Litsea* (Himalaya, Western Ghats), *Polygala sibirica* (Himalaya, Khasi Hills, Western Ghats and Sri Lanka), *Rhamnus virgatus* (Himalaya, Palni and Tinnevelly Hills), *Stellaria media* (Himalaya, Nilgiri, Shevaroy, Palni and Sri Lanka), *Thalictrum* (Himalaya and Anaimalai Hills), *Viburnum acuminatum* (Mahendragiri, Shevaroy, Palni and Nilgiri Hills), and *Viola patrinii* (Himalaya, Mahendragiri, Shevaroy, Nilgiri and Palni Hills) (see Mani, 1974).

## Present-day Himalayan Biodiversity

The present-day Himalayan ecosystems exhibit a pronounced post-Pleistocene biodiversity, but retain the mixed biotic character of having immigrant elements from different biogeographic regions. Biogeographically, the Himalaya is a transitional zone located at the cusp of three biogeographic realms, namely Palearctic, Afrotropical and Indo-Malayan and Oriental realms in the north, southwest and southeast, respectively (see Mani, 1974). As a result of this unique geographic location, the Himalaya is home to numerous Austro-Polynesian, Sino-Tibetan, Euro-Mediterranean and Malayo-Burman biotic elements. A simplified plant biodiversity profile occupying different Himalayan zones, viz. Western, Central and Eastern Himalaya is presented in Table 2. The elevational limits of different vegetation zones are slightly higher by 300–400 m in the Eastern Himalaya than the Western Himalaya (Pandit, 2017). The principal reason for such an elevational zone shift is the latitudinal difference of nearly 10° between the Eastern and Western Himalaya (see Pandit, 2017).

**Table 2:** Generalized vegetation profile of the Western, Central and Eastern Himalaya. Overall, Western Himalaya is characterized by coniferous forests of deodar, pines and silver fir, while Eastern Himalaya shows conspicuous presence of broad-leaved forests of oaks, Rhododendrons and maples (Source: Adapted from Pandit & Kumar, 2013; Pandit, Manish & Koh, 2014; Manish et al., 2017; Pandit, 2017).

**Himalayan zone**	**Climate zones**		
	Tropical and sub-tropical	Temperate	Sub-alpine and alpine
Western	Semi-deciduous forests of *Shorea robusta*, *Acacia catechu*, *Dalbergia sissoo*, *Albizia lebbeck*, *Garuga pinnata*, *Terminalia bellirica* and *T. tomentosa* are found up to 1,500 m; at higher elevations, *Pinus roxburghii* occurs	This zone (1,500–3,500 m) is dominated by oaks (*Quercus* spp.) and *Rhododendron* spp. *Cedrus deodara*, *Abies pindrow* and *Picea smithiana* dominate elevations between 2,800–3,500 m	This zone (3,500 m and above) show preponderance of herbaceous genera of *Anemone*, *Geranium*, *Iris*, *Lloydia*, *Potentilla*, *Primula* etc. interspersed with dry dwarf alpine scrubs of *Berberis*, *Cotoneaster*, *Juniperus* and *Rhododendron*
Central	*Shorea robusta*, *Acacia catechu* and *Dalbergia sissoo* comprise principal tree species of this zone up to 1,500 m along with *Haldina cordifolia*, *Kydia calycina* and *Semecarpus anacardium*; *Pinus roxburghii* forests appear at higher elevations	Forest vegetation is similar to Western Himalaya, albeit with lesser number of *Rhododendron* species and with additional presence of *Berberis* spp. and *Prinsepia utilis*	
Eastern	*Mesua assamica*, *Mesua ferrea*, *Albizia procera*, *Bombax ceiba*, *Careya arborea*, *Gmelina arborea*, *Oroxylum indicum*, *Duabanga grandiflora* dominate up to 1,800 m; at higher elevations, *Quercus lamellosa* and *Mesua* forests are found	Evergreen oak forests of *Quercus lamellosa* and *Q. cerris* dominate along with *Magnolia* spp., *Lithocarpus pachyphyllus* and *Acer* spp.; between 2,800–3,600 m, *Abies delavayi*, *Abies densa*, *Tsuga dumosa*, *Larix griffithii* and *Rhododendron* dominate	

The development of present-day plant biodiversity in the Himalaya has largely been shaped by the climate of the region. In the tropical and sub-tropical zones (up to 1,500 m), temperature varies from 6 °C to 35 °C in various areas while rainfall variation is from 1,500 to 3,500 mm. These climate conditions are well suited for growth of deciduous and semi-deciduous vegetation. The temperate zone (1,500–3,500 m) experiences an average annual rainfall of 2,400 mm while temperature varies between 5–26 °C. Thus, mainly coniferous forests dominate the temperate zone in the Himalaya. Low temperatures characterize the sub-alpine and alpine zones (3,500 m and above) and the precipitation takes place mainly in the form of snowfall, except in the summer, which lasts for only three months. Thus, only plant communities with specialized adaptations to these harsh conditions can survive in the sub-alpine and alpine zones, such as cushion forming communities (*Arenaria polytrichoides*, *Anaphalis cavei*), tussocks or tufts (*Kobresia schoenoides*, *Carex parva*), solifluction acrobats (*Gentiana urnula*, *Eriophytum wallichii*), and dense woolly forms (*Saussurea graminifolia*, *Glechoma nivalis*).

## Conclusions

The geophysical upheavals associated with the formation of the Himalaya led to significant climate changes, new geophysical environments, novel ecological niches and formation of physical and physiological isolation barriers that acted as natural selection sieves. These geophysical changes including onset of monsoon, glaciation and glacial advance, and retreat brought about adaptive radiations in the plant taxa. The Himalayan landforms were initially colonized by migrant plant taxa from the neighboring biogeographic regions and in due course, these early taxa established gene exchanges among them and formed new variants that were highly adaptive to the changing physiography and climate of the area. Once the final phase of the Himalayan uplift concluded, the environmental conditions became suitable for the migrant species and the variants to evolve and diversify into new taxa. Disjunct taxa distributions were brought about by the fragmented landscape, while new physical and physiological barriers limited the expansion of species ranges. Newly formed steep gorges and valleys imposed isolation barriers on the recently migrated species enhancing the processes and rates of evolutionary divergence. As a net result, many endemic taxa evolved and the Himalaya became a repository of unique assemblages of plant species. Thus, having started as a ‘sink’ and a ‘biological highway’, the Himalaya turned into a geographical barrier, promoting vicariance, species diversification and endemism. However, only a few studies have empirically tested the above hypotheses and those that have are based on only specific taxa. It is well known that different patterns of diversification may exist for different taxa. Therefore, many of the stated hypotheses here may appear as conjectures. Many unanswered questions still remain, that include: (i) what was the nature of early migrants in the Himalaya; (ii) what were the ancestral distributional areas of these migrants; (iii) what were the precise time periods during which maximum migrations and endemic diversification occurred in the Himalaya; and (iv) were the migrations and diversifications widespread across phylogenies? A complementary and integrative collaboration between researchers from varied backgrounds in earth sciences, atmospheric sciences, palaeobiology, biogeography and ecology are needed to solve these evolutionary riddles in an important global biodiversity hotspot—the Himalaya.

## Additional Information and Declarations

### Data Availability

The following information was supplied regarding data availability: Since this is a Literature Review, the research in this article did not generate any data or code. All the references have been cited in the text itself.
